# Antioxidant Intervention Attenuates Aging-Related Changes in the Murine Ovary and Oocyte

**DOI:** 10.3390/life10110250

**Published:** 2020-10-22

**Authors:** Mandy G. Katz-Jaffe, Sydney L. Lane, Jason C. Parks, Blair R. McCallie, Rachel Makloski, William B. Schoolcraft

**Affiliations:** Colorado Center for Reproductive Medicine, Lone Tree, CO 80124, USA; SLane@ColoCRM.com (S.L.L.); JParks@ColoCRM.com (J.C.P.); BMcCallie@ColoCRM.com (B.R.M.); RMakloski@ColoCRM.com (R.M.); BSchoolcraft@ColoCRM.com (W.B.S.)

**Keywords:** antioxidants, flavonoids, oxidative stress, advanced maternal age, in vitro fertilization, dietary supplements

## Abstract

Advanced maternal age (AMA) is associated with reduced fertility due in part to diminished ovarian follicle quantity, inferior oocyte quality, chromosome aneuploidy, and lower implantation rates. Ovarian aging is accompanied by increased oxidative stress and blunted antioxidant signaling, such that antioxidant intervention could improve reproductive potential. The first aim of this study was to determine the molecular effects of antioxidant intervention in the ovaries and oocytes of aged mice, utilizing a supplement containing only naturally occurring açaí (Euterpe oleracea) with an oxygen radical absorbance capacity of 208,628 μmol Trolox equivalent (TE)/100 g indicating high antioxidant activity. Nine month old female CF-1 mice were administered 80 mg/day antioxidants (*n* = 12) or standard diet (*n* = 12) for 12 weeks. In the ovary, antioxidant treatment upregulated β-adrenergic signaling, downregulated apoptosis and proinflammatory signaling, and variably affected cell growth and antioxidant pathways (*p* < 0.05). Exogenous antioxidants also increased the oocyte expression of antioxidant genes *GPX1*, *SOD2*, and *GSR* (*p* < 0.05). A feasibility analysis was then conducted on female AMA infertility patients as a proof-of-principle investigation. Patients (*n* = 121; <45 years old) consented to receiving 600 mg antioxidants three times daily for ≥8 weeks preceding infertility treatment. Preliminary results indicate promising outcomes for AMA patients, warranting further investigation.

## 1. Introduction

Advanced maternal age (AMA) represents a significant decrease in fertility associated with reduced ovarian follicle quantity and oocyte quality, increased oocyte chromosome aneuploidy, and lower implantation rates [1,2,3]. Aging is associated with an imbalanced redox state in most organs, including the ovaries, with increased reactive oxygen species (ROS) relative to antioxidant signaling [4,5]. In aged ovaries, this is likely due in part to less activity of naturally occurring antioxidant enzymes [6,7]. Transcriptional changes also contribute to aging effects in a variety of ways. Specifically, antioxidant gene expression decreases in mouse, nonhuman primate, and human oocytes [8,9,10,11], altered microRNAs in human blastocysts lead to reduced oxidative defense [12], and granulosa and cumulus cell gene expression important for oocyte maturation is dysregulated [11,13,14]. Aged oocytes are also more sensitive to oxidative stress and likely require a shift in redox balance toward increased antioxidant signaling [15].

The relationship between ROS and antioxidant signaling in the ovary is complex. Ovarian ROS are important mediators of numerous processes including ovulation, corpus luteum formation, and ovarian angiogenesis [16,17,18,19], but they can also be detrimental to ovarian processes such as cumulus–oocyte complex interactions and oocyte maturation [20,21]. Therefore, the correct balance between ROS generation and elimination is essential for optimal ovarian function and fertility [22]. The aging-associated decline in fertility has shown correlation with dysregulated pro- and antioxidant signaling [11,23,24]. Studies utilizing various animal models of induced ovarian oxidative stress provide evidence that antioxidant supplementation combats oxidative damage in the ovary [25,26,27,28,29,30].

In patients with increased levels of oxidative stress, clinical studies have linked positive pregnancy outcomes with more robust antioxidant signaling. One such study in patients with unexplained infertility found that a higher intake of certain antioxidant nutrients was associated with decreased time to pregnancy [31]. Another study observed a positive correlation between the total antioxidant response measured in maternal blood and pregnancy success following in vitro fertilization (IVF) [32]. Consistent with these studies, elevated circulating or peritoneal ROS are associated with idiopathic infertility [33,34]. Therefore, we hypothesize that augmenting antioxidant signaling may improve ovarian function and fertility.

The primary aim of this study was to determine the effects of antioxidant treatment on gene expression in ovaries and oocytes of aged mice. We utilized the naturally occurring açaí berry (*Euterpe oleracea*) as it contains flavonoids that are potent scavengers of oxygen free radicals and ROS, and it shows therapeutic benefit in several studies investigating aging impact [35,36]. Dietary antioxidant supplementation was sufficient to alter gene expression in both ovaries and oocytes. Signaling pathways that are impacted by aging, i.e., antioxidant and apoptosis, were altered by antioxidant treatment. We then evaluated the feasibility of antioxidant administration in patients prior to an IVF cycle and determined that patients were compliant with the antioxidant regimen, where it was found that reproductive outcomes in AMA and younger counterparts were comparable. We conclude that antioxidant supplementation is a feasible and promising strategy to improve ovarian function during reproductive aging, warranting further investigation.

## 2. Results

### 2.1. Pathway Analysis of the Aged Murine Ovarian Transcriptome Following Antioxidant Intervention

**Figure 1:**
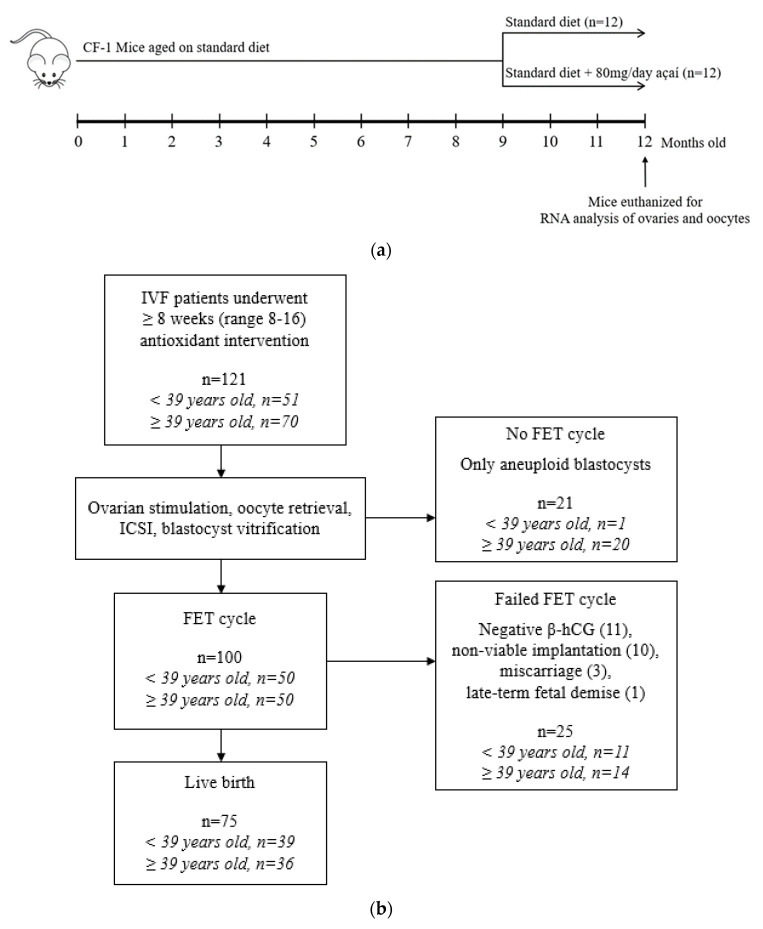
Experimental designs for antioxidant intervention in (**a**) mice and (**b**) humans are detailed.

**Figure 2:**
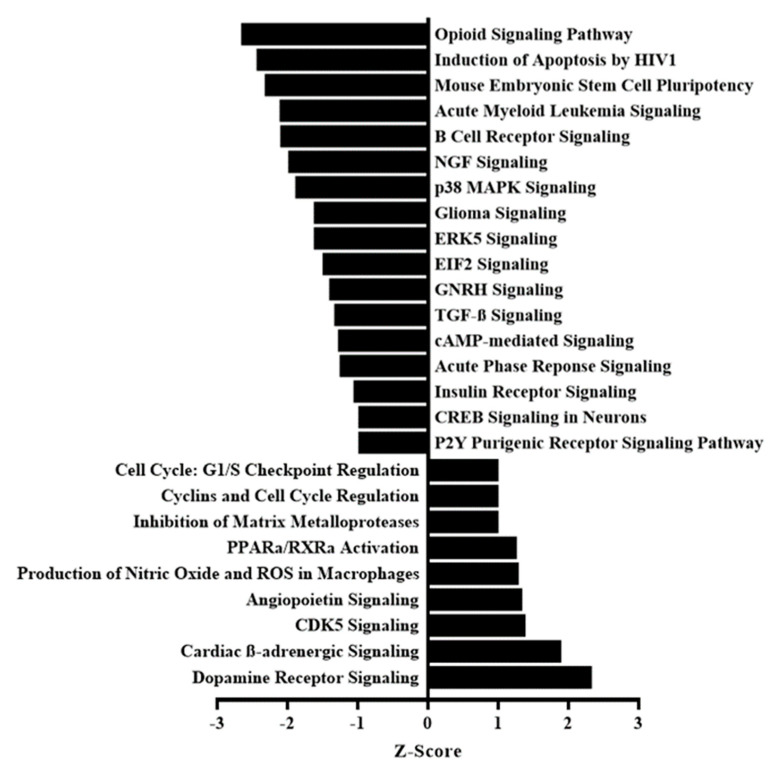
Pathway analysis of the differentially expressed genes in aged murine ovaries following antioxidant intervention. Cellular pathways with altered expression after antioxidant intervention compared to control diet, with *p* < 0.05 and *Z*-score >1 or <−1, are presented.

After 9 months of natural aging, female mice were fed either a control chow diet or the same diet supplemented with antioxidants for 12 weeks, after which they were euthanized for collection of ovaries and oocytes (Figure 1a). Murine ovarian transcriptome analysis revealed that, compared to a control diet, antioxidant treatment altered the expression of many signaling pathways in the ovaries of aged mice as determined by pathway analysis (Figure 2) of the differentially expressed genes (Table S1, Supplementary Materials). Antioxidant administration resulted in upregulated β-adrenergic signaling (dopamine receptor, cardiac β-adrenergic, and cyclin-dependent kinase 5 (CDK5) signaling pathways). Apoptosis signaling was downregulated (induction of apoptosis via the human immunodeficiency virus 1 (*HIV1*) pathway) with reduced expression of tumor necrosis factor receptor superfamily member 1B (*TNFRSF1B*). Cell growth pathways were both upregulated (cyclins and cell-cycle regulation, gap 1 (G1)/synthesis (S) checkpoint regulation, and angiopoietin signaling pathways), and downregulated (transforming growth factor beta (TGFβ) and p38 mitogen-activated protein kinase (MAPK) signaling pathways), suggesting that antioxidant treatment promoted cell-type-specific cell survival and growth.

Antioxidant intervention had anti-inflammatory effects by downregulating proinflammatory pathways (p38 MAPK and B-cell receptor signaling pathways; nuclear factor (NF)-κB signaling, *Z*-score −0.632). The intervention also had antioxidant effects per microarray findings, i.e., increased expression of the antioxidant gene *PAX4*, but did not result in a significant difference by pathway analysis.

### 2.2. Differential Gene Expression in the Murine Ovary Following Antioxidant Intervention

**Figure 3:**
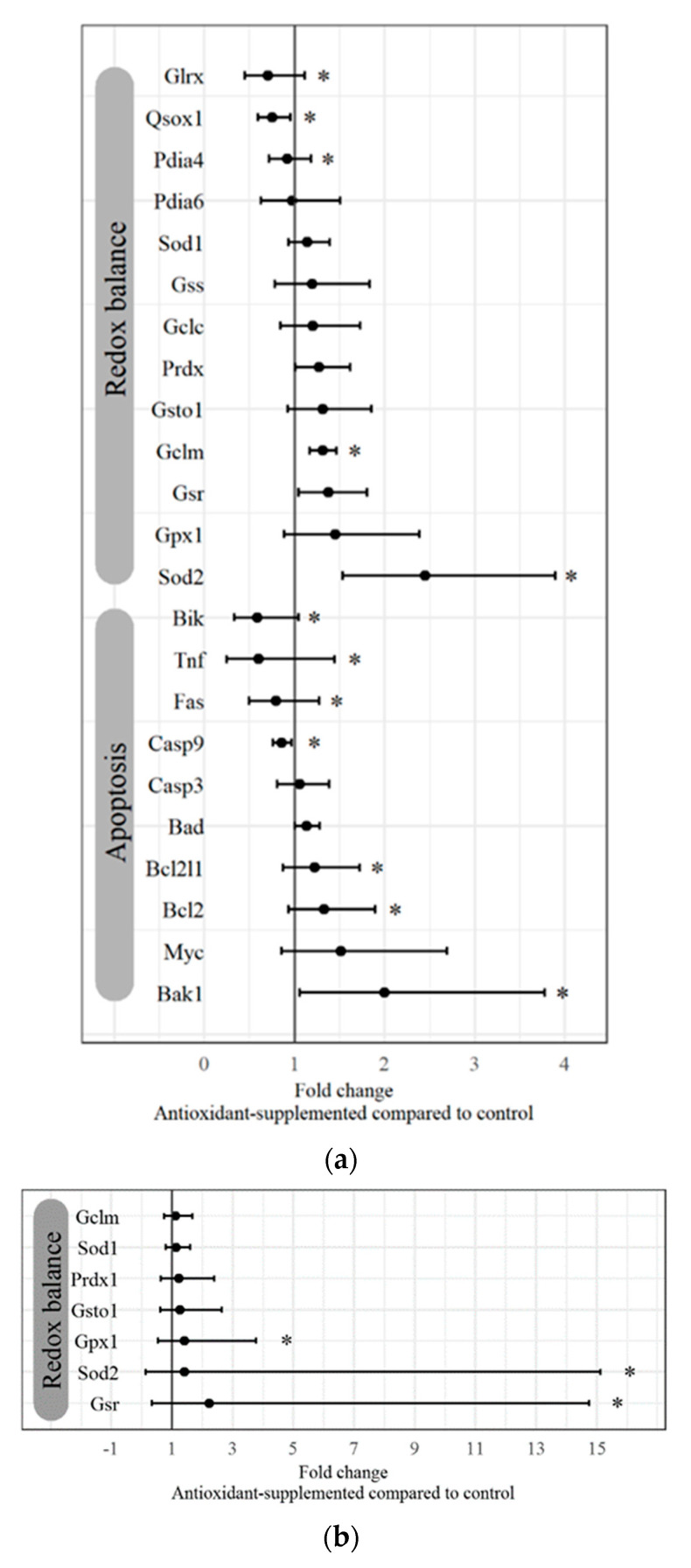
Antioxidant-induced gene expression changes in the aged murine ovary and meiosis II (MII) oocytes. Select genes identified as differentially expressed by microarray and additional genes of interest were analyzed by qPCR. Data are presented as the fold change (± fold change range) of gene expression in (**a**) ovaries (*n* = 12 each) and (**b**) MII oocytes (*n* = 19 each) from antioxidant-treated aged mice compared to aged mice on control diet. * *p* < 0.05 by two-tailed, unpaired *t*-test.

Effects of antioxidant treatment on apoptosis signaling in the ovary were further investigated by qPCR (Figure 3a), and the microarray and qPCR findings were consistent. Expression of tumor necrosis factor (*TNF*)-related genes was lower after antioxidant treatment as determined by both methodologies. The microarray did not report levels of the other genes analyzed by qPCR. Differentially expressed genes by qPCR reflected a shift toward pro-survival and antiapoptotic signaling. Antioxidant intervention downregulated the extracellular-induced apoptotic signaling response, with reduced expression of proapoptotic factors *TNF* and Fas cell-surface death receptor (*FAS*). Intrinsically induced apoptosis was also downregulated by antioxidant treatment, with reduced expression of the proapoptotic factor Bcl-2-interacting killer (*BIK*) and higher expression of pro-survival factors Bcl2-like 1 (*BCL2L1*), B-cell lymphoma 2 apoptosis regulator (*BCL2*), and Bcl-2 homologous antagonist killer (*BAK1*). Consistently, expression of the proapoptosis regulator caspase 9 (*CASP9*) was reduced. 

We determined by qPCR that ovarian signaling components important for redox balance were variably affected by antioxidant treatment, with reduced expression of the antioxidant enzymes glutaredoxin (*GLRX*), quiescin sulfhydryl oxidase 1 (*QSOX1*), and protein disulfide isomerase family A member 4 (*PDIA4*), but increased expression of the antioxidant genes glutamate cysteine ligase modifier subunit (*GCLM*) and superoxide dismutase 2 (*SOD2*) (Figure 3a).

### 2.3. Effects of Antioxidant Administration on Murine Oocyte Gene Expression

Antioxidant intervention altered the gene expression of factors important in redox signaling in murine oocytes (Figure 3b). Expression levels of three important antioxidant genes—glutathione peroxidase (*GPX1*), *SOD2*, and glutathione reductase (*GSR*)—were markedly higher in oocytes from mice treated with antioxidants compared to control diet.

### 2.4. Feasibility Analysis after Antioxidant Intervention

To determine feasibility of the antioxidant intervention in a patient cohort, infertility patients were administered antioxidants for 8–16 (mean 9.9 ± 2.2) weeks (variability due to menstrual cycle dates), immediately preceding follicle-stimulating hormone (FSH) administration (Figure 1b). Patients were aged 38.1 ± 3.5 (median 39, range 28–44) years during their antioxidant-supplemented IVF cycle. The patients had normal ovarian reserves as determined by measurement of antral follicle count and follicle-stimulating hormone (FSH) on menstrual cycle day 3 and antimüllerian hormone levels (Table 1). In total, 121 patients were offered the treatment, and all consented and were compliant.

**Figure 4:**
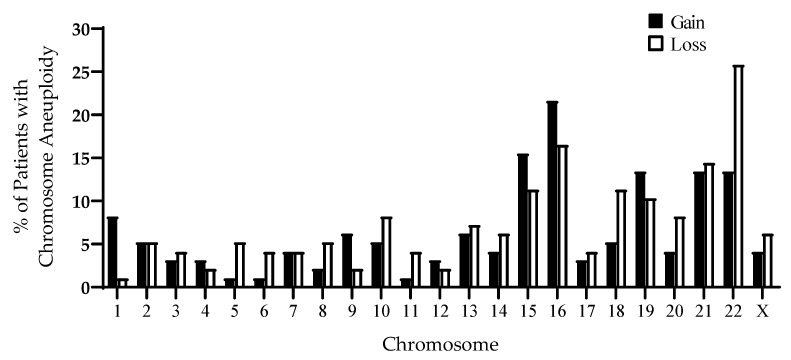
Types of aneuploidies in blastocysts from antioxidant-treated patients (*n* = 553 blastocysts, 100 IVF cycles). The percentage of patients with a gain or loss of each chromosome is presented.

Preimplantation genetic testing for aneuploidy (PGT-A) was performed by array comparative genomic hybridization (aCGH) or next-generation sequencing (NGS; Illumina, San Diego, CA, USA) for detection of whole-chromosome aneuploidy only. Trisomies of chromosomes 15, 16, 19, 21, and 22 and monosomies of chromosomes 15, 16, 18, 19, 21, and 22 each occurred in more than 10% of patients (Figure 4). In total, 21 patients had only aneuploid blastocysts and did not undergo frozen embryo transfer (FET; Figure 1b). The IVF cycles that resulted in only aneuploid blastocysts occurred in women who were significantly older than patients who underwent FET (41.5 ± 1.7 vs. 37.4 ± 3.4 years old, *p* < 0.0001). In patients 39 years or older compared to patients younger than 39, IVF outcomes were similar apart from euploidy rates, which were lower in the older group (*p* < 0.0001; Table 1). 

### 2.5. Outcomes of FET Following Antioxidant Supplementation

All subjects underwent follow-up after FET procedures, and 75% resulted in live birth (Table 1). In total, 99.3% of cryopreserved embryos survived warming and 1.4 ± 0.5 embryos were transferred per patient according to the physician’s direction and their prior infertility history. Cases of reproductive failure included 11 negative β-human chorionic gonadotropin (β-hCG) measurements, seven biochemical pregnancy losses, three pregnancies resulting in first-trimester fetal loss (no products of conception analyzed), and one late-gestation fetal demise due to a placental defect. AMA patients 39 years or older had promising FET outcomes comparable to good-prognosis patients younger than 39 (Table 1).

## 3. Discussion

Antioxidant supplementation in aged mice was sufficient to impact the ovarian and oocyte transcriptome, specifically, signaling pathways affected by aging. Infertility patients were compliant with the antioxidant regimen prior to an IVF cycle and AMA patients had promising and comparable reproductive outcomes to their younger counterparts. These findings support our hypothesis that reduced antioxidant signaling in the aged ovary can be ameliorated by exogenous antioxidant intervention to enhance reproductive potential.

Following antioxidant administration, the murine ovarian transcriptome reflected an environment with enhanced cell survival and β-adrenergic signaling, decreased apoptotic signaling proinflammatory pathways, and altered antioxidant signaling compared to control aged mice. Upregulated β-adrenergic signaling may contribute to improved ovarian function through changes in follicular development and hormone secretion [37,38,39], particularly since ovarian sympathetic nerve activity may decline with ovarian aging [40]. The anti-inflammatory effects of antioxidant treatment are consistent with previous reports [41]. Further cell-type-specific investigation into the effects of antioxidant treatment on redox regulation in the ovary is warranted, given that some antioxidant enzymes were upregulated while others were downregulated. A recently published study of nonhuman primates provided insight into ovarian cell-type-specific changes in gene expression with aging [11] and reported increased apoptosis and reduced antioxidants in the ovary, which they localized to granulosa cells. The aged mice in this study showed a similar phenotype to aged nonhuman primates, and antioxidant intervention was sufficient to alter the cellular processes most influenced by aging, i.e., apoptosis and antioxidant signaling. In the whole ovary, the expression of important antioxidant genes was upregulated, including *GCLM*, the modifier subunit of glutamate cysteine ligase (GCL). Decreased activity of GCL is associated with increased oxidative stress and aging-related diminished function in the ovary [42]; thus, we speculate that increased *GCLM* expression reduces oxidative stress and improves ovarian function.

In murine oocytes, expression of key antioxidant genes *SOD2*, *GPX1*, and *GSR* was markedly higher after treatment. Disrupted redox balance associated with aging reduces oocyte competence [22] such that increased expression of these genes in oocytes of antioxidant-supplemented mice could lead to restoration of quality and future developmental competence. Superoxide dismutase is important in meiosis for maintenance of sister chromatid cohesion necessary to prevent segregation errors and aneuploidy [43,44]; thus, its upregulation following antioxidant intervention could reduce meiotic errors. Glutathione peroxidase activation in oocytes has also been associated with enhanced embryonic development [45,46]. The importance of increasing antioxidant enzyme expression in oocytes was highlighted by a recent study that reported downregulation of these genes (including *GPX1* and *GSR*) with aging in the oocytes of nonhuman primates [11]. Restoration of redox balance in the oocyte may be beneficial for maintaining correct epigenetic signatures which are important for euploidy, fertilization potential, and embryo development [47].

On the basis of these observations, infertility patients were recruited for a proof-of-principle investigation of antioxidant supplementation prior to an IVF cycle to analyze its feasibility. In AMA patients 39 years of age or older compared to their younger counterparts, IVF and FET outcomes were similar with the exception of aneuploidy rates that were higher for the AMA patients, as extensively documented in the literature [48]. The 21 patients who did not undergo FET due to aneuploidy were significantly older than the antioxidant-supplemented patients who underwent FET (41.5 ± 1.7 vs. 37.4 ± 3.4 years old, *p* < 0.0001). There was a lack of euploidy in 17.4% of patients, which is in the expected range for women of this age (36.7 ± 3.7 years old, *n* = 121) [49]. Thus, antioxidants did not negate the known effect of advanced maternal aging as women enter their 40s as the most significant risk factor for chromosome aneuploidy. The antioxidant regimen was not detrimental to IVF outcomes, as live birth rates were comparable to typical rates at our clinic and even represent possible improvements [50]. The preliminary findings of this proof-of-principle investigation provide rationale for future studies on the efficacy of antioxidant intervention.

Our findings may have broader implications for antioxidant administration in additional populations. For example, there was elevated anti-inflammatory gene expression in the ovary with antioxidants, suggesting that patients with pathologic ovarian inflammation may benefit from antioxidant treatment. In women with obesity and/or polycystic ovary syndrome, there is evidence that chronic, low-grade inflammation and oxidative stress in both ovarian follicles and the endometrium contribute to anovulation and implantation failure, indicating that these groups may benefit from this intervention [51,52].

There is robust evidence that an imbalanced redox state contributes to the effects of aging, and that oxidative stress and aging are associated with lower fertility. So far, there is a lack of knowledge of the benefits of antioxidant treatment in the preconception period on assisted reproductive technology outcomes. In an aged murine model, antioxidant treatment positively impacted ovarian and oocyte signaling pathways impacted by aging. When the antioxidant supplement was administered to AMA patients prior to an IVF cycle, reproductive outcomes were similar to those in younger counterparts. Antioxidant supplementation is a promising strategy to achieve optimal ovarian function and oocyte quality, warranting further investigation.

## 4. Materials and Methods 

### 4.1. Murine Antioxidant Intervention Protocol

Female outbred CF-1 mice (Charles River, Wilmington, MA, USA) were housed with a 12 h light/dark cycle and ad libitum access to standard chow diet and water. All animal procedures and protocols were completed in accordance with the Guide for the Care and Use of Laboratory Animals (8th edition) and approved by the Fertility Labs of Colorado Ethics in Research Committee. Mice were naturally aged for 9 months prior to an antioxidant diet intervention of 80 mg açaí daily for 12 weeks (*n* = 12; Figure 1a). The açaí antioxidant supplement was sourced from a commercial manufacturer and contained only the naturally occurring açaí berry from the palm tree *Euterpe oleracea*. The açaí pulp underwent nonthermal dehydration and packaging into vegetable-based cellulose capsules (Ecofruits International Inc., South Jordan, UT, USA). Chemical analysis of the lot by the manufacturer reported a total polyphenol content of 6618 mg gallic acid equivalent (GAE)/100 g, an oxygen radical absorbance capacity (ORAC) of 208,628 μmol Trolox equivalent (TE)/100 g, and negligible microbial contamination. Control mice (*n* = 12) received the same balanced nutritional feed but without the açaí supplementation. After 12 weeks of the antioxidant intervention or control diet, mice were euthanized by cervical dislocation for immediate collection of ovaries or oocytes. 

For oocyte collection, mice were superovulated with 5 IU pregnant mare’s serum gonadotropin (Sigma G-4877, St. Louis, MO, USA) followed 48 h later by 5 IU hCG (Sigma CG-5). Then, 23 h after hCG administration, animals were euthanized by cervical dislocation, and oviducts were immediately dissected from the abdominal cavity. Cumulus oocyte masses were then isolated from the ampullae in G-MOPS+ (Vitrolife, Stockholm, Sweden) and exposed to hyaluronidase (Sigma H-3757) to denude the cumulus cells and isolate the oocytes for collection.

### 4.2. Murine RNA Isolation, Transcriptome Analysis, and Quantitative Real-Time PCR

Murine ovaries were harvested from 12 month old mice, and RNA was isolated using the RNeasy mini kit after homogenization with the QiaShredder (Qiagen, Germantown, MD, USA). RNA analysis was carried out with CodeLink™ Mouse Whole Genome Array (Applied Microarrays, Tempe, AZ, USA) and Ingenuity Pathway Analysis (Qiagen). 

RNA was isolated from oocytes in meiosis II (MII) using the PicoPure™ RNA Isolation Kit (Applied Biosystems, Foster City, CA, USA) with minor modifications to the manufacturer’s protocol. Briefly, oocytes were lysed at 42 °C for 30 min in 10 μL of extraction buffer. One volume of 70% ethanol was mixed with each sample prior to loading onto a preconditioned purification column. Each sample was on-column deoxyribonuclease-treated at room temperature for 15 min (Qiagen). After several washes, RNA was eluted in 20 μL of elution buffer.

For microarray validation and further gene expression investigation in ovaries and MII oocyte gene expression, reverse transcription was performed using the High-Capacity complementary DNA (cDNA) Reverse Transcription Kit (Applied Biosystems). The same extracted RNA samples were used for both ovarian microarray and gene expression. The cDNA was diluted (1:4) in 1× Tris–ethylenediaminetetraacetic acid (EDTA) buffer prior to performing quantitative reverse-transcription PCR (RT-qPCR) on the 7300 Real-Time PCR System (Applied Biosystems). Here, 3 μL of diluted cDNA was combined with 5 μM primer mix and Power SYBR™ Green PCR Master Mix (Applied Biosystems) in a 15 μL final volume and amplified under the following thermal cycling conditions: 95 °C for 10 min, followed by 40 cycles at 95 °C for 15 s and 60 °C for 1 min, and a melt curve stage at 95 °C for 15 s, 60 °C for 1 min, and 95 °C for 15 s. Gene primers are described in Table S2 (Supplementary Materials). For each target gene, relative expression to the stable internal housekeeping gene ribosomal protein L19 (*RPL19*) was determined for sample-to-sample comparisons. The fold change in expression of antioxidant-supplemented vs. control mice was determined using the 2^−ΔΔCT^ method.

### 4.3. Feasibility Analysis of Antioxidant Intervention Protocol and Outcomes

A total of 121 patients undergoing IVF with autologous oocytes consented to participate in this proof-of-principle investigation. All female patients were <45 years and their partners had no severe male factor infertility or requirement for surgically removed sperm. Patients provided informed consent (Health One Institutional Review Board, protocol no. 350763-4) and were not compensated for their participation.

The intervention protocol is described in Figure 1b. Routine IVF protocols were followed as described previously [53]. Patients were administered antioxidants—the same açaí supplements described above—orally in 600 mg capsules three times daily according to the variable reported doses of açaí pulp administered [54], and compliance was assessed by subjects’ verbal confirmation. The duration of intervention was 8–16 (mean 9.9 ± 2.2) weeks (variability due to menstrual cycle dates), immediately preceding FSH administration. Standard protocols were followed for ovarian stimulation, IVF, and PGT-A. 

The primary patient outcomes were indicators of ovarian function and oocyte quality, i.e., the number of oocytes retrieved, blastocyst development, and blastocyst euploidy. The patients underwent follow-up to determine subsequent reproductive outcomes.

### 4.4. Statistical Analysis

Murine microarray data were analyzed by Ingenuity Pathway Analysis (Qiagen) and pathways with *p* < 0.05 and *Z*-score >1 or <−1 are reported. Murine qPCR data are presented as fold change compared to control ± the fold change range. Statistical analysis was performed using REST 2009 software (Qiagen)m which uses PCR efficiencies and mean crossing point deviation between the sample and control groups to test for significance via a Pair-Wise Fixed Reallocation Randomization Test^©^ (significance at *p* < 0.05).

## Figures and Tables

**Table 1:** Results of proof-of-principle investigation.

	FET Patients <39 Years, *n* = 50	FET Patients ≥39 Years, *n* = 50
Age, years	34.5 ± 2.4	40.3 ± 1.3
Prior failed IVF cycles, *n* (range)	2.1 ± 1.4 (1–8)	1.9 ± 1.4 (1–7)
**Baseline ovarian reserve parameters**		
AMH, ng/mL	2.9 ± 2.9	2.8 ± 2.1
D3 FSH, ng/mL	8.0 ± 3.4	8.4 ± 3.1
D3 AFC, *n*	16.6 ± 8.3	15.7 ± 8.5
**IVF outcomes**		
Oocytes collected, *n*	17.4 ± 8.3	17.4 ± 9.2
Total blastocysts, *n*	5.0 ± 2.9	5.2 ± 3.3
Total blastocysts, %	30.4 ± 14.1	33.7 ± 18.3
Euploid blastocysts, *n*	3.0 ± 1.9	2.1 ± 1.6
Euploid blastocysts, %	63.8 ± 24.8	43.4 ± 22.3 *
Embryos transferred	1.5 ± 0.5	1.4 ± 0.5
**FET outcomes**		
Implantation rate (FHT)	72.0%	75.3%
Negative β-hCG	4 (8%)	7 (14%)
Nonviable implantation	6 (12%)	4 (8%)
Miscarriage	1 (2%)	2 (4%)
Fetal demise at 34 weeks	0 (0%)	1 (2%)
Live birth	39 (78%)	36 (72%)
Twin birth	11 (28%)	9 (24%)

FET, frozen embryo transfer; β-hCG, β-human chorionic gonadotropin; AMH, antimüllerian hormone; FSH, follicle-stimulating hormone; AFC, antral follicle count; D3, menstrual cycle day 3; FHT, fetal heart tones; IVF, in vitro fertilization. Data are presented as means ± SD or number (%) of patients. * *p* < 0.0001 by two-tailed, unpaired *t*-test.

## Supplementary Materials

The following are available online at https://www.mdpi.com/2075-1729/10/11/250/s1: Table S1. Differentially expressed genes in aged murine ovarian tissue after antioxidant supplementation (*n* = 12); Table S2. Primer sequences for quantitative real-time polymerase chain reaction of ovarian and oocyte genes.

## Abbreviations

AMA	Advanced maternal age
ROS	Reactive oxygen species
IVF	In vitro fertilization
CASP9	Caspase 9
CDK5	Cyclin-dependent kinase 5
HIV1	Human immunodeficiency virus 1
TNFRSF1B	TNF receptor superfamily member 1B
TGFβ	Transforming growth factor beta
MAPK	Mitogen-activated protein kinase
NF-κB	Nuclear factor kappa light chain enhancer of activated B cells
PAX4	Paired box gene 4
TNF	Tumor necrosis factor
FAS	Fas cell surface death receptor
BIK	Bcl-2-interacting killer
BCL2L1	Bcl-2-like 1
BCL2	B-cell lymphoma 2 apoptosis regulator
BAK1	Bcl-2 homologous antagonist killer
GLRX	Glutaredoxin
QSOX1	Quiescin sulfhydryl oxidase 1
PDIA4	Protein disulfide isomerase family A member 4
GCLM	Glutamate cysteine ligase modifier subunit
SOD2	Superoxide dismutase 2
GPX1	Glutathione peroxidase
GSR	Glutathione reductase
FSH	Follicle stimulating hormone
FET	Frozen embryo transfer
PGT-A	Preimplantation genetic testing for aneuploidy
aCGH	Array comparative genomic hybridization
NGS	Next generation sequencing
β-hCG	Beta-human chorionic gonadotropin
GAE	Gallic acid equivalent
ORAC	Oxygen radical absorbance capacity
TE	Trolox equivalent
RPL19	Ribosomal protein L19
