# Supplementary material for: Antioxidant Intervention Attenuates Aging-Related Changes in the Murine Ovary and Oocyte
Source: Life (Basel). 2020 Oct 22;10(11):250. doi: 10.3390/life10110250 (PMC7690403; doi:10.3390/life10110250)
Supplement: Supplementary file 1 [file life-10-00250-s001.zip › life-950780-supplementary.docx]

Supplementary Materials of Antioxidant Intervention Attenuates Aging-Related Changes in the Murine Ovary and Oocyte

**Table S1.** Differentially expressed genes in aged murine ovarian tissue after antioxidant supplementation (n = 12).

| **Gene** | **Control Gene Dxpression** | **+Antioxidant Gene Expression** | **Fold Change** | ***P* Value** |
| --- | --- | --- | --- | --- |
| Bpil3 | 8.44 | 64.3 | 2.93 | 0.003 |
| Kcnj16 | 2.85 | 17.1 | 2.58 | 0.043 |
| Cpa3 | 1.54 | 8.79 | 2.52 | 0.013 |
| 2610028H24Rik | 1.60 | 8.76 | 2.45 | 0.023 |
| 1600029D21Rik | 4.30 | 22.7 | 2.40 | 0.020 |
| 2010001J22Rik | 2.15 | 10.9 | 2.34 | 0.004 |
| AU041480 | 1.74 | 7.44 | 2.10 | 0.039 |
| Zmynd10 | 1.43 | 6.01 | 2.08 | 0.024 |
| E030011K20Rik | 4.31 | 16.6 | 1.95 | 0.048 |
| A2m | 2.02 | 7.67 | 1.92 | 0.043 |
| 1700003G18Rik | 1.68 | 6.19 | 1.88 | 0.047 |
| D15Bwg0759e | 2.28 | 8.27 | 1.86 | 0.025 |
| Zbed3 | 4.82 | 16.6 | 1.79 | 0.019 |
| Dsp | 4.33 | 14.8 | 1.78 | 0.020 |
| 5730446C15Rik | 5.41 | 18.4 | 1.76 | 0.013 |
| BB288505 | 1.17 | 3.73 | 1.68 | 0.004 |
| Stab2 | 1.84 | 5.77 | 1.65 | 0.007 |
| Slc9a3r1 | 7.84 | 24.3 | 1.63 | 0.020 |
| Card4 | 2.41 | 6.95 | 1.53 | 0.019 |
| Ndufa5 | 3.04 | 8.61 | 1.50 | 0.025 |
| Ecm1 | 57.7 | 161 | 1.48 | 0.026 |
| Cd300lg | 1.90 | 5.22 | 1.46 | 0.014 |
| 2700055K07Rik | 6.64 | 18.1 | 1.44 | 0.031 |
| Slc7a5 | 4.53 | 12.2 | 1.43 | 0.023 |
| Ela1 | 5.46 | 14.4 | 1.40 | 0.010 |
| 4930544G21Rik | 5.14 | 12.9 | 1.33 | 0.026 |
| Slc6a7 | 1.14 | 2.80 | 1.30 | 0.011 |
| Birc1e | 11.9 | 28.7 | 1.28 | 0.035 |
| Ccl19 | 5.15 | 12.3 | 1.26 | 0.047 |
| Dbp | 13.8 | 33.1 | 1.26 | 0.011 |
| Kmo | 0.840 | 2.00 | 1.25 | 0.034 |
| Ankrd47 | 3.54 | 8.34 | 1.24 | 0.009 |
| Gm836 | 7.08 | 16.3 | 1.21 | 0.012 |
| 4933432I09Rik | 2.95 | 6.75 | 1.20 | 0.027 |
| Nfe2 | 1.72 | 3.93 | 1.20 | 0.033 |
| Tmem68 | 6.62 | 15.1 | 1.19 | 0.000 |
| Il10ra | 0.945 | 2.15 | 1.19 | 0.006 |
| Plekhf1 | 6.79 | 15.3 | 1.17 | 0.006 |
| Rarres2 | 18.2 | 40.8 | 1.16 | 0.007 |
| Dusp14 | 4.35 | 9.69 | 1.16 | 0.001 |
| Fmo3 | 1.07 | 2.36 | 1.14 | 0.025 |
| Glra4 | 3.56 | 7.85 | 1.14 | 0.036 |
| Commd6 | 4.34 | 9.50 | 1.13 | 0.043 |
| Arpp19 | 7.54 | 16.4 | 1.12 | 0.038 |
| Bbox1 | 8.12 | 17.5 | 1.11 | 0.016 |
| Mcpt6 | 6.98 | 15.0 | 1.10 | 0.034 |
| Lsm5 | 3.53 | 7.55 | 1.10 | 0.023 |
| Il1f10 | 0.937 | 2.00 | 1.10 | 0.036 |
| Tnfaip8l3 | 3.54 | 7.54 | 1.09 | 0.002 |
| Ces5 | 3.30 | 7.04 | 1.09 | 0.006 |
| Rpl39 | 148 | 314 | 1.09 | 0.009 |
| Tmem23 | 7.29 | 15.3 | 1.07 | 0.029 |
| Elovl1 | 9.98 | 21.0 | 1.07 | 0.043 |
| Polr3gl | 3.80 | 7.95 | 1.07 | 0.002 |
| Mapk15 | 1.80 | 3.72 | 1.05 | 0.009 |
| Atf3 | 5.13 | 10.6 | 1.05 | 0.032 |
| Zfyve9 | 3.29 | 6.76 | 1.04 | 0.010 |
| Aak1 | 9.78 | 20.0 | 1.03 | 0.044 |
| Saa3 | 15.4 | 31.4 | 1.03 | 0.031 |
| Ddx47 | 2.29 | 4.67 | 1.03 | 0.002 |
| Gup1 | 0.995 | 2.02 | 1.02 | 0.009 |
| Cryba4 | 2.40 | 4.87 | 1.02 | 0.013 |
| Cml4 | 1.42 | 2.86 | 1.01 | 0.016 |
| Slc12a2 | 2.27 | 4.58 | 1.01 | 0.002 |
| Rexo1 | 3.70 | 1.84 | -1.01 | 0.004 |
| Slc16a3 | 12.4 | 6.13 | -1.01 | 0.013 |
| Dact2 | 6.12 | 3.01 | -1.02 | 0.034 |
| Gm496 | 2.03 | 0.996 | -1.02 | 0.005 |
| Fip1l1 | 15.6 | 7.66 | -1.03 | 0.001 |
| Laptm5 | 7.64 | 3.75 | -1.03 | 0.006 |
| Pgf | 1.79 | 0.876 | -1.03 | 0.020 |
| 4930572J05Rik | 8.13 | 3.97 | -1.03 | 0.024 |
| Adh1 | 84.5 | 41.3 | -1.03 | 0.012 |
| Dbx1 | 1.63 | 0.794 | -1.03 | 0.000 |
| Speer4d | 3.16 | 1.52 | -1.05 | 0.033 |
| Stambp | 12.0 | 5.66 | -1.08 | 0.006 |
| AL033314 | 17.1 | 7.97 | -1.11 | 0.021 |
| Ly6a | 44.1 | 20.5 | -1.11 | 0.002 |
| Itsn1 | 5.75 | 2.66 | -1.11 | 0.014 |
| Ear2 | 3.10 | 1.41 | -1.14 | 0.011 |
| LOC545794 | 1.70 | 0.769 | -1.15 | 0.040 |
| Fn1 | 8.96 | 4.01 | -1.16 | 0.008 |
| Glul | 96.6 | 43.2 | -1.162 | 0.019 |
| Dst | 3.17 | 1.41 | -1.167 | 0.004 |
| 9130017N09Rik | 9.86 | 4.37 | -1.174 | 0.017 |
| Smarca1 | 11.7 | 5.14 | -1.181 | 0.024 |
| Gpbp1l1 | 9.63 | 4.24 | -1.182 | 0.029 |
| Sema3d | 3.83 | 1.67 | -1.195 | 0.005 |
| Pabpc4 | 6.53 | 2.84 | -1.201 | 0.013 |
| Psmb2 | 70.6 | 30.7 | -1.205 | 0.000 |
| Inpp4a | 2.77 | 1.19 | -1.217 | 0.002 |
| Prkacb | 2.81 | 1.20 | -1.225 | 0.004 |
| Agr2 | 2.91 | 1.24 | -1.228 | 0.019 |
| 2600005C20Rik | 6.18 | 2.62 | -1.235 | 0.003 |
| Ctsb | 12.4 | 5.24 | -1.247 | 0.001 |
| Cd72 | 2.04 | 0.860 | -1.249 | 0.008 |
| Cspg2 | 12.7 | 5.23 | -1.277 | 0.024 |
| Ppp2r3a | 9.21 | 3.73 | -1.305 | 0.014 |
| 2210412D01Rik | 11.0 | 4.29 | -1.361 | 0.012 |
| Fcna | 4.38 | 1.68 | -1.385 | 0.039 |
| Fbxo11 | 6.91 | 2.64 | -1.386 | 0.001 |
| Chchd3 | 30.8 | 11.8 | -1.388 | 0.034 |
| Strm | 3.43 | 1.30 | -1.404 | 0.016 |
| Mgat4c | 2.24 | 0.833 | -1.430 | 0.002 |
| Herpud2 | 4.03 | 1.48 | -1.448 | 0.004 |
| AI481105 | 26.2 | 9.45 | -1.468 | 0.024 |
| 1110007F05Rik | 11.9 | 4.23 | -1.490 | 0.028 |
| Ms4a4b | 4.56 | 1.61 | -1.502 | 0.008 |
| Ly6k | 5.66 | 1.99 | -1.509 | 0.034 |
| Ikbkb | 10.7 | 3.60 | -1.578 | 0.011 |
| Xpnpep2 | 5.41 | 1.68 | -1.691 | 0.047 |
| Ctsc | 18.4 | 5.60 | -1.713 | 0.011 |
| Olfr558 | 2.72 | 0.728 | -1.903 | 0.021 |
| Cdkl1 | 7.81 | 2.02 | -1.950 | 0.014 |
| 9430016A21Rik | 8.01 | 2.06 | -1.963 | 0.036 |
| Gpr173 | 8.94 | 2.10 | -2.090 | 0.038 |
| H2-Q10 | 17.5 | 4.10 | -2.090 | 0.017 |
| Enah | 8.50 | 1.83 | -2.214 | 0.007 |
| Mrpl21 | 22.5 | 4.76 | -2.244 | 0.036 |
| 2610528J11Rik | 11.5 | 2.42 | -2.245 | 0.032 |
| 2310033K02Rik | 4.53 | 0.862 | -2.395 | 0.001 |
| Spdef | 5.04 | 0.950 | -2.407 | 0.029 |
| Comp | 7.74 | 1.42 | -2.442 | 0.011 |
| Il17rb | 7.48 | 1.33 | -2.487 | 0.041 |
| Tcf3 | 5.04 | 0.808 | -2.640 | 0.014 |
| Slc1a1 | 11.5 | 1.78 | -2.686 | 0.036 |
| Irx1 | 6.56 | 0.996 | -2.718 | 0.002 |
| B4galt1 | 16.5 | 2.35 | -2.808 | 0.010 |
| Sftpd | 13.7 | 1.65 | -3.051 | 0.029 |
| Rab25 | 23.6 | 2.83 | -3.062 | 0.018 |
| Slc14a1 | 17.5 | 2.06 | -3.083 | 0.009 |
| 9230116B18Rik | 9.89 | 1.06 | -3.225 | 0.005 |
| Pllp | 15.1 | 1.39 | -3.447 | 0.011 |
| Gdpd3 | 24.2 | 1.94 | -3.639 | 0.011 |
| 9930023K05Rik | 22.5 | 1.74 | -3.695 | 0.006 |
| Tmc5 | 12.7 | 0.851 | -3.901 | 0.002 |
| *Genes with fold change >1.0 or <-1.0 and P<0.05 compared to control are presented.* | | | | |

**Table S2.** Primer sequences for quantitative real-time polymerase chain reaction of ovarian and oocyte genes.

| **Gene** | **Primer Sequence (5’-3’)** | **Genbank Accession** |
| --- | --- | --- |
| Glrx | F: CCTGCATTCACTGCCCTTAC  R: GCGGGTTGTAGGAGGTTGA | NM_053108 |
| Qsox1 | F: GACCGCTCCAAGATCTACATG  R: CCCACTTCTACACGCAAGATG | NM_023268 |
| Pdia4 | F: GTGGAGAGGATGTCAATGCA  R: TGGCTCCATGGCAAACTTC | NM_009787 |
| Sod1 | F: CAGAAGGCAAGCGGTGAAC  R: CATGCTGGCCTTCAGTTAATCC | NM_011434 |
| Gss | F: CGGTGGTGCTACTGATTGC  R: CGGCACGCTGGTCAAATA | NM_008180 |
| Gclc | F: GGCCACTATCTGCCCAATTG  R: CACGTAGCCTCGGTAAAATGG | NM_010295 |
| Prdx | F: TGGCTCGACCCTGCTGATA  R: TTGAAGTTGGGAGCAGGATACC | NM_011034 |
| Gsto1 | F: AGCGACTGGAAGCATTGG  R: CGCCATCCAGAGCTTGAG | NM_010362 |
| Gclm | F: CCAGATTTGACTGCCTTTGCTA  R: GAGCAGTTCTTTCGGGTCATTG | NM_008129 |
| Gsr | F: GCTCCACACATCCTGATTGC  R: TCTGGCTCTCGTGAGGAACTG | NM_010344 |
| Gpx1 | F: CAGCAGTCTGGCAACTCCTAAG  R: GAGTGCAGCCAGTAATCACCAA | NM_008160 |
| Sod2 | F: TAGGGCCTGTCCGATGATG  R: TGAATGGCTTCCCAGAATGC | NM_013671 |
| Bik | F: AGGGTGTTCGGGCAGTTC  R: ATAAGTCTCGCCTCCGACATG | NM_007546 |
| Tnf | F: GGCAGGTTCTGTCCCTTTCAC  R: TCGCGGATCATGCTTTCTG | NM_013693 |
| Fas | F: ACTGCACCCTGACCCAGAATAC  R: CAGTGTTCACAGCCAGGAGAATC | NM_007987 |
| Casp9 | F: AAGTGGCTCCTGGTACATCGA  R: GCATTGGCAACCCTGAGAAG | NM_015733 |
| Casp3 | F: GGTTCATCCAGTCCCTTTGC  R: CTGTTAACGCGAGTGAGAATGTG | NM_009810 |
| Bad | F: TGCAACACAGATGCGACAAAG  R: GGGATGTGGAGCAGAAGATCAC | NM_007522 |
| Bcl2l1 | F: TGTGCGTGGAAAGCGTAGAC  R: GGCCATCCAACTTGCAATC | NM_009743 |
| Bcl2 | F: TGTGTGGAGAGCGTCAACAG  R: GATGCCGGTTCAGGTACTCA | NM_009741 |
| Myc | F: GTCTTTCCCTACCCGCTCAAC  R: GTGGAATCGGACGAGGTACAG | NM_010849 |
| Bak1 | F: AGCACCATGAATCCACTGATACC  R: GCCGTGCAAAGACGAAGAC | NM_007523 |

**Publisher’s Note:** MDPI stays neutral with regard to jurisdictional claims in published maps and institutional affiliations.

| 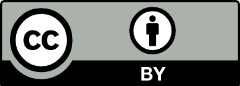 | © 2020 by the authors. Submitted for possible open access publication under the terms and conditions of the Creative Commons Attribution (CC BY) license (http://creativecommons.org/licenses/by/4.0/). |
| --- | --- |
